# Comparing the efficacy of dual Platelet-Rich Plasma (PRP) and Hyaluronic Acid (HA) therapy with PRP-alone therapy in the treatment of knee osteoarthritis: a systematic review and meta-analysis

**DOI:** 10.1186/s40634-021-00415-1

**Published:** 2021-11-04

**Authors:** Angeline Ai Ling Aw, Jun Jie Leeu, Xinyu Tao, Hamid Rahmatullah Bin Abd Razak

**Affiliations:** 1 Lee Kong Chian School of Medicine, Nanyang Technological University, 59 Nanyang Drive, Experimental Medicine Building, Singapore, 636921 Singapore; 2 Department of Orthopaedic Surgery, Sengkang General Hospital, 110 Sengkang East Way, Singapore, 544886 Singapore; 3 SingHealth Duke-NUS Musculoskeletal Sciences Academic Clinical Programme, 20 College Road, Academia Level 4, Singapore, 169865 Singapore

**Keywords:** Platelet-rich plasma, Hyaluronic acid, Knee osteoarthritis

## Abstract

**Purpose:**

This study aims to compare the efficacy of a dual therapy of Platelet-Rich Plasma (PRP) and Hyaluronic Acid (HA) compared with PRP-alone therapy in the treatment of knee osteoarthritis (KOA).

**Methods:**

PubMed, Embase, CINAHL, SCOPUS, Cochrane Library, grey literature and bibliographic references were searched from inception to January 2021. Only randomized controlled trials (RCTs) and retrospective cohort studies comparing the effect of PRP and HA versus PRP-alone therapy for KOA were included. Literature retrieval and data extraction were conducted by three independent reviewers. Pooled analysis of Visual Analogue Scale (VAS), Western Ontario and McMaster Universities Arthritis Index (WOMAC), International Knee Documentation Committee (IKDC) scores and adverse events were conducted.

**Results:**

Ten studies (7 RCTs, 3 cohort studies) involving 983 patients were covered. Dual PRP and HA therapy resulted in significant reduction in VAS compared to PRP-alone therapy at 4–6 weeks (*P* < 0.00001) and 12 months (*P* < 0.00001). Dual therapy resulted in better WOMAC score improvement at 3 (*P* = 0.02), 6 (*P* = 0.05) and 12 months (*P* < 0.0001) compared to PRP-alone therapy. The IKDC score for dual therapy was also higher at 6 months compared to PRP-alone therapy (*P* = 0.007). Regarding adverse events, dual therapy was generally safer than PRP-alone therapy (*P* = 0.02).

**Conclusion:**

While there is a paucity of large high-quality Level I studies, current best evidence suggests that dual therapy with PRP and HA for KOA may be effective at providing pain relief and improvement in function up to 1 year following administration.

**Level of evidence:**

II.

## Introduction

Knee osteoarthritis (KOA) is the most diagnosed form of osteoarthritis, accounting for almost four-fifths of the global burden of OA and resulting in debilitating pain and loss of function [9]. The incidence of KOA has continued to grow over the past few decades with no signs of slowing down, in line with the global phenomenon of longevity and ageing populations [43]. Hence, KOA is presently a huge burden on healthcare systems and societies globally [9].

Currently, there is no effective cure for KOA apart from knee arthroplasty which is usually offered at an advanced stage of disease [2]. The focus of many clinicians today lies in the prevention and treatment of the disease in the early stage to prevent any further progression. The American Academy of Orthopaedic Surgeons recommends conservative treatment methods before progressing to surgical intervention when they fail [20]. Conservative treatment, which can be broadly categorised into pharmacological and nonpharmacological methods, aims to retain patient mobility and reduce pain. Non-pharmacological treatment includes preventive and rehabilitative measures such as weight loss and diet control as KOA is highly related to obesity [37]. Such measures, being heavily dependent on patient compliance, are often out of the jurisdiction of clinicians and are often used concurrently with pharmacological treatment such as oral analgesics and non-steroidal anti-inflammatory drugs [37]. Pharmacological modalities have seen varying success depending on the stage of the disease, and despite their efficacy in controlling the disease in its early stages and slowing its progression, these drugs come with various contraindications and side effects.

Clinicians have also adopted the use of intra-articular injection of hyaluronic acid (HA) and platelet-rich plasma (PRP), both individually and synergistically, to treat KOA. PRP, generally defined as autologous plasma with a higher concentration of platelets compared to peripheral blood, is obtained by centrifugation of autologous blood [5]. The release of growth factors and cytokines after platelet degranulation is thought to promote healing and reduce inflammation, leading to it being widely used in other musculoskeletal diseases [11]. HA is a naturally occuring fluid which provides lubrication and aids in shock absorption [39]. Similar to PRP, it is also shown to have anti-inflammatory effects on cells in vitro [4]. Systematic reviews have concluded that while both modalities, used individually, resulted in improved clinical outcomes by alleviating pain and slowing the progress of KOA, patients who received PRP experience better outcomes compared to HA [7, 19].

In recent years, studies have been exploring the feasibility of combining HA and PRP as a dual therapy. Some studies have found that using these two drugs simultaneously might be beneficial to patients through synergistic biochemical mechanisms [42]. However, there has been a paucity of systematic reviews and meta-analyses evaluating whether combination therapy results in significantly improved clinical outcomes compared to PRP-alone therapy. This systematic review compares patient-reported outcomes on pain, stiffness and function to provide a methodical analysis of the available literature on the efficacy of PRP and HA versus PRP-alone therapy in the treatment of KOA.

## Methods

This systematic review and meta-analysis was conducted in accordance with relevant requirements of the Preferred Reporting Items for Systematic Reviews and Meta-Analyses (PRISMA) Statement.

### Literature search strategy

In order to retrieve relevant literature, different databases were perused; MEDLINE (PubMed), Embase, Cumulative Index to Nursing and Allied Health (CINAHL), Cochrane Library, SCOPUS, grey literature (conference proceedings, industry white papers, Google Scholar) and bibliographic references were hand-searched to identify relevant studies. Out of the relevant studies, only randomized controlled trials (RCTs) and retrospective cohort studies were selected and followed up on. This retrieval period spanned from establishment of each database to September 2021. Three researchers (AA, JJ, XY) cross-referenced information on Covidence to reduce data extraction errors. Search terms engaged consisted of (Hyaluronic Acid OR Hyaluronan OR Hyaluronate Sodium OR Sodium Hyaluronate) AND Knee AND (Osteoarthritis OR Degenerative OR Arthroses OR Arthrosis OR Osteoarthrosis OR Osteoarthrosis Deformans) AND (Platelet-rich Plasma OR Autologous Protein Solution OR Autologous Conditioned Serum OR Autologous Conditioned Plasma OR Platelet-derived Growth Factors OR Platelet-Rich Fibrin OR L-PRF).

### Inclusion and exclusion criteria

Inclusion criteria for the study include the following: (1) Type of study: Published RCTs or retrospective cohort studies, (2) Research subjects: Individuals diagnosed with KOA, regardless of age, gender, or nationality, (3) Intervention: Intra-articular injection dual therapy of PRP combined with HA versus PRP-alone therapy, (4) Outcomes: At least one of the following outcome indicators cited: Western Ontario and McMaster Universities Arthritis Index (WOMAC) score, International Knee Documentation Committee (IKDC) score, or visual analogue scale (VAS), (5) Studies written in English or Chinese language.

Exclusion criteria for the study include the following: (1) Type of study: Retrospective studies, reviews, case reports, and case series, (2) Research Subjects: Subjects with non-knee osteoarthritis, animal subjects or cadavers, (3) Intervention: Studies in which the intervention did not include intra-articular injection of PRP combined with HA, (4) Outcomes: Studies detailing the mechanism of PRP and HA, (5) Studies not written in English or Chinese language, (6) Studies with data that are unable to be extracted, (7) Studies with data that do not report mean and standard deviation.

### Study selection and data extraction

The literature retrieval was conducted under the guidelines of established inclusion and exclusion criteria. Two reviewers (AA, JJ) extracted each set of data independently before compilation and cross-referencing on Covidence. A third researcher (XY) and corresponding author assisted in the cross-referencing process independently to minimize judgment errors.

The quantitative data extracted in this study included first author, publication year, sample size, intervention measures, ethical approval, gender, age, BMI, follow-up periods, Kellgren-Lawrence radiographic classification, relevant items for literature quality evaluation, WOMAC, IKDC, VAS scores and adverse events.

### Quality assessment of included studies

Regarding RCTs, the Cochrane Risk of Bias Tool was employed for quality evaluation. The tool includes evaluation in seven domains: (1) random sequence generation (selection bias), (2) allocation concealment (selection bias), (3) blinding of participants and personnel (performance bias), (4) blinding of outcome (detection bias), (5) incomplete outcome data (attrition bias), (6) selective reporting (reporting bias), and (7) other sources of bias. The risk of bias in each domain was judged to be low, high or unclear.

For the cohort studies, the Risk Of Bias In Non-randomized Studies of Interventions (ROBINS-I) tool was used for quality assessment. This tool evaluates bias in seven domains: (1) bias due to confounding, (2) bias in selection of participants into the study, (3) bias in classification of interventions, (4) bias due to deviations from intended interventions, (5) bias due to missing data, (6) bias is measurement of outcomes, and (7) bias in selection of the reported result. The risk of bias in each domain was judged to be low, moderate, serious or critical.

With regards to publication bias, the included studies were reviewed for declaration of potential publication bias. The risk of bias in this domain was recorded to be either present, or absent.

Quality assessment of the studies was performed independently by three reviewers (AA, JJ, XY) and any differences were resolved by consensus.

### Statistical analysis

To evaluate our main outcome of comparing the efficacy of PRP and HA with PRP-alone therapy, a fixed-effects model was employed for the meta-analysis, with the only exception being the comparison of VAS scores at baseline. The mean difference (MD) was used to evaluate the effects of continuous variables comparing the three different types of output (IKDC, WOMAC, and VAS), with 95% confidence intervals (CIs) of MD being calculated. Review Manager 5.4 software (Cochrane Collaboration, Oxford, UK) was used to calculate the efficacy and safety indicators and their 95% CIs. A *P* value less than 0.05 was judged as statistically significant.

## Results

### Literature screening process and results

**Fig. 1:**
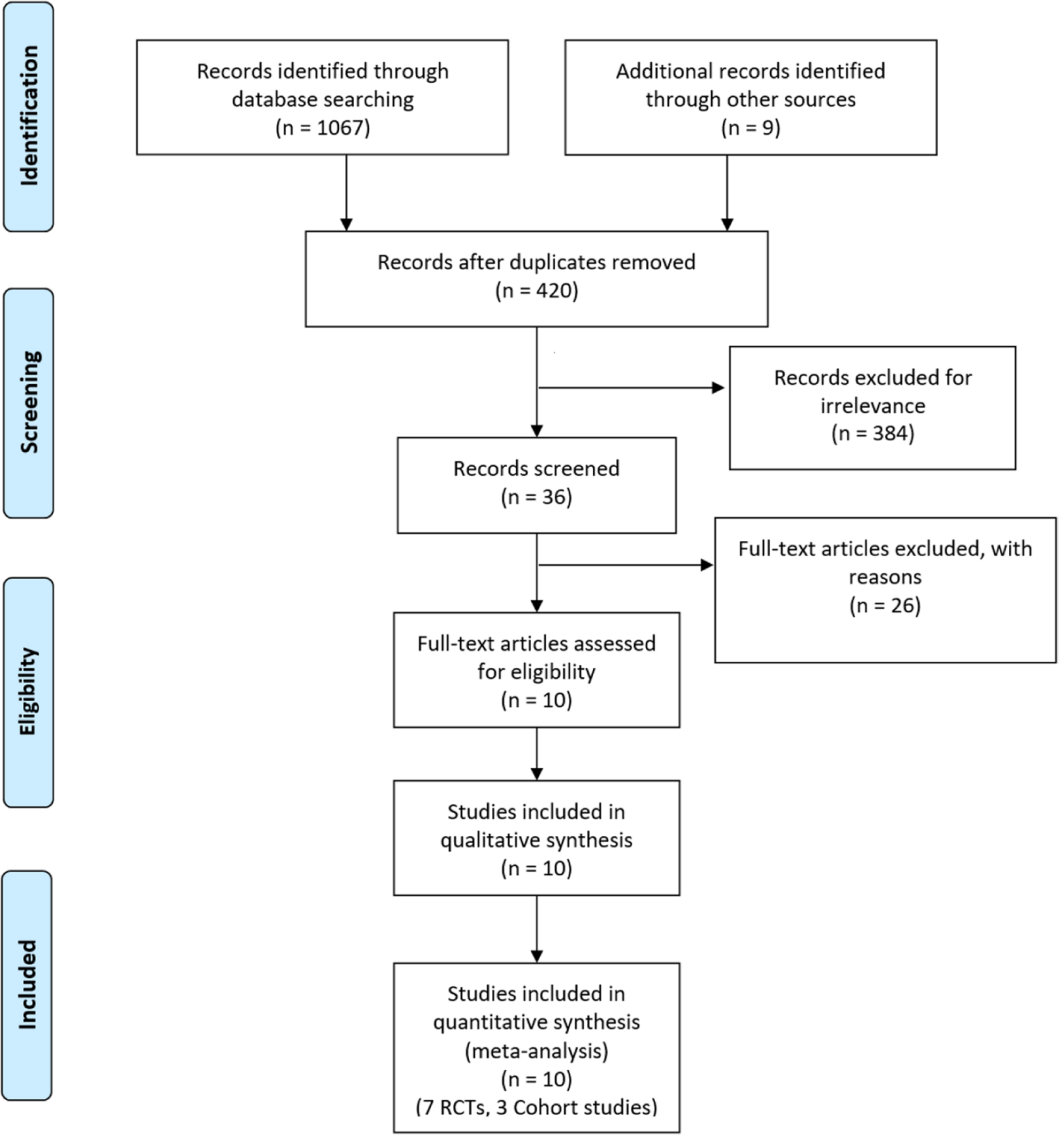
Showing the PRISMA flowchart of the study search process

A preliminary examination of titles and abstracts yielded a total of 420 relevant studies, after the removal of duplicates. Following the guidelines of strict inclusion and exclusion criteria, the final sample size of ethically approved studies was narrowed down to ten, involving seven RCTs [1–4, 7–8, 10) and three retrospective cohort studies (5–6, 9). These studies hold a total patient sample size of 983. Seven were two-arm studies, and three three-arm studies. The literature screening process and results are reflected in Fig. 1. Age of patients included in this study ranged from 22 to 78 years, Kellgren and Lawrence radiographic grading scale of KOA ranged from I to IV, and the follow-up period spanned 6–12 months (Table 1). Baseline demographic data such as age, gender, and BMI, and sample size of the patients included in the 8 studies were comparable (*p* = n.s.). It is important to note that the 2017 study by Jacob et al. [21] was analysed separately due to the usage of 2 formulations of PRP: HMW and LMW. Duration of treatment ranged from 3 to 8 weeks and the interval between injections ranged from 1 to 2 weeks (Table 2). All the studies administered the PRP and HA in the PRP + HA arm concomitantly, except for Ke et al., who administered 4 ml of PRP first then 2 ml of HA 10 min later, and Lana et al., who administered 2 ml of HA first then 5 ml of PRP.

**Table 1:** detailing the characteristics of the included studies

First Author, Year	Study design	Language	Total number of patients	Number of patients		Age		Gender (F:M)		Kellgren-Lawrence Score (I:II:III:IV)		Follow-up period	Outcomes reported
				PRP + HA	PRP	PRP + HA	PRP	PRP + HA	PRP	PRP + HA	PRP		
**Ding, 2017** [12]	RCT	Chinese	47	27	20	56.75 ± 9.54	62.11 ± 12.5	18:2	19:8	9:6:5:0	10:11:6:0	6 months	VAS, WOMAC, Lequesne
**Zhao, 2018** [45]	RCT	Chinese	124	62	62	55.73 ± 7.18	56.32 ± 8.13	27:35	25:37	–	–	5 weeks	VAS, JOA
**Ke, 2016** [22]	RCT	Chinese	150	50	50	57.80 ± 6.90	59.30 ± 7.10	25:25	26:24	5:11:20:14	6:12:20:12	12 months	WOMAC, IKDC, Lequesne
**Guo, 2018** [17]	RCT	Chinese	126	63	63	61.00 ± 10.00	61.00 ± 10.00	18:45	12:51	17:28:18:0	15:31:17:0	12 months	VAS, WOMAC
**Abate, 2015** [1]	Retrospective cohort	English	80	40	40	56.70 ± 11.20	60.90 ± 9.00	9:31	19:21	0:23:17:0	0:19:21:0	6 months	VAS, KOOS
**Guo, 2016** [18]	Retrospective cohort	English	126	63	63	61.20 ± 9.60	60.70 ± 10.10	45:18	51:12	17:28:18:0	15:31:17:0	12 months	VAS, WOMAC
**Jacob, 2017** [21]	RCT	English	51	20	31	–	–	–	–	–	–	6 months	VAS, IKDC
**Yu, 2018** [42]	RCT	English	360	104	96	46.50 ± 7.50	46.20 ± 8.60	46:50	54:50	–	–	12 months	WOMAC
**Palco, 2021** [28]	Retrospective cohort	English	51	28	23	62.71 ± 7.88	54.04 ± 10.4	16:12	11:12	0:8:20:0	0:10:13:0	12 months	VAS, KSS, KOOS
**Sun, 2021** [37]	RCT	English	78	39	39	60.6 ± 8.4	58.4 ± 8.1	18:21	22:17	0:39:0:0	0:39:0:0	6 months	VAS, WOMAC, Lequesne, SLS

**Table 2:** detailing the details of the administration and composition of PRP + HA and PRP alone of the included studies. NS = not specified

First Author, Year	Study Type	Procedural Details	PRP + HA composition (ml)	PRP Composition (ml)	Number of injections	Injection Interval (weeks)
**Ding, 2017** [12]	RCT	**PRP**: 40 ml venous blood added to sodium citrate; centrifuged for 10 min at 1450 rpm; PCP aspirated and transferred out, then centrifuged for 10 min at 3370 rpm; yield 5 ml PRP **HA**: 25 mg/2.5 ml PRP and HA administered together	PRP: 4 HA: 2.5	4	3	1
**Zhao, 2018** [45]	RCT	**PRP**: 5 ml whole blood centrifuged to obtain PRP PRP and HA administered together	PRP: 4 HA: 2.5	2	5	1
**Ke,** **2016** [22]	RCT	**PRP**: 40 ml venous blood added to 4 ml of sodium citrate; centrifuged for 10 min at 1500 rpm; PCP aspirated and transferred out, then centrifuged for 10 min at 1500 rpm; activation with 0.2 ml of calcium chloride; yield 4 ml PRP PRP administered first, HA 10 min later	PRP: 4 HA: 2	6	5	1
**Guo, 2018** [17]	RCT	**PRP**: 45 ml blood added to 5 ml of citrate phosphate dextrose; centrifuged for 10 min at 2000 rpm; PCP aspirated and transferred out, then centrifuged for 10 min at 2200 rpm; yield 7 ml PRP PRP and HA administered together	PRP: 3.5 HA: 2	3.5	3	1
**Abate, 2015** [1]	Retrospective cohort	**PRP+ HA**: 4 ml blood added to 2 ml HA (natural, non-crosslinked, fermented, 1550 kDa, 40 mg); centrifuged for 5 min at 3500 rpm; yield 2 ml of PRP + 2 ml of HA **PRP**: 8 ml blood centrifuged for 5 min at 3500 rpm; yield 4-5 ml PRP and HA administered together	PRP: 2 HA: 2	4–5	3	1
**Guo, 2016** [18]	Retrospective cohort	NS PRP and HA administered together	PRP: NS HA: NS	NS	3	1
**Jacob, 2017** [21]	RCT	**PRP**: 20-ml venous blood added to 5-ml of citrate phosphate dextrose; centrifuged for 7 min at 3500 rpm; PCP aspirated and transferred out, then centrifuged for 5 min at 3000 rpm; yield 2.5 ml PRP Administration details NS	PRP: NS HA: NS	2	NS	NS
**Yu,** **2018** [42]	RCT	**PRP**: Plasma samples were prepared immediately using centrifugation at 2000 g at 4 °C for 10 min PRP and HA administered together	PRP: 8 HA: 0.2 mg	8	8	1
**Palco, 2021** [28]	Retrospective cohort	**PRP + HA**: CellularMatrix A-CP-HA used; 8-ml of venous blood centrifuged for 5 min at 3400 rpm/1500 g; yield 3 ml PRP; Platelet concentration: 290 k platelets/mm3 **L-PRP**: RegenKit-THT-3/RegenCell used; 8-ml of venous blood centrifuged for 9 min at 3400 rpm/1500 g; yield 5 ml PRP; Platelet concentration: 340 k platelets/mm3 Administration details NS	PRP: 3 HA: 2	5	NS	NS
**Sun, 2021** [37]	RCT	**PRP**: 7-mL of venous blood collected in PLTenus PLUS Platelet Concentrate Separator; centrifuged at 500-1200 rpm for 8 min; yield 3 ml LP-PRP; Average platelet count: 463.83 ± 75.39 × 103/ul **HA**: HYAJOINT Plus; cross-linked with BDDE 3 ml of HA injected first, then 3 ml of PRP	PRP: 3 HA: 3	3	1	NS

### Quality assessment of the included literature

**Fig. 2:**
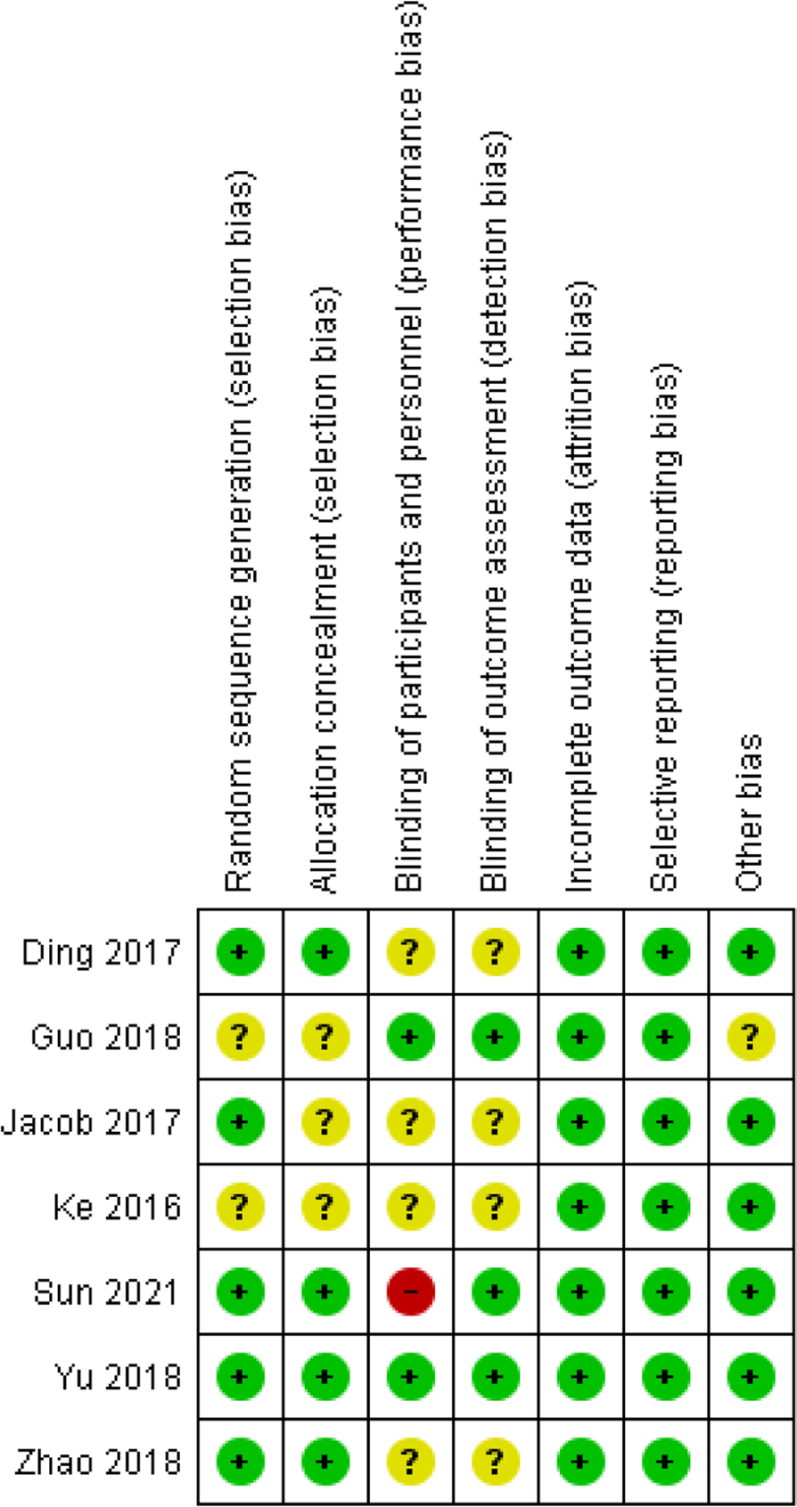
Detailing the quality assessment of the 7 RCTs using the Cochrane ROB tool

**Fig. 3:**
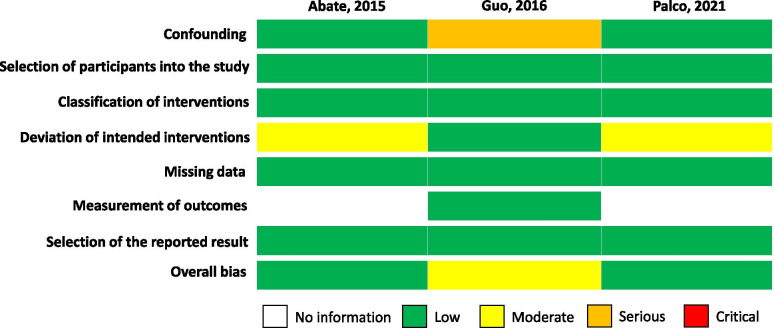
Detailing the quality assessment of the 3 cohort studies using the ROBINS-I tool. Green: Low risk, Yellow: Moderate risk, Orange: Serious risk, Red: Critical risk

The overall methodological quality of the included studies is summarized in Figs. 2 and 3.

For the RCTs, only one study (Sun 2021) had a high risk of performance bias as the injector and patients were not blinded in the study (Fig. 2). For random sequence generation, five (71%) studies were at low risk of bias, while two (29%) studies were of unclear risk. For allocation concealment, four (57%) studies were at low risk of bias, while three (43%) studies were of unclear risk. Studies such as Guo 2018 [17] mentioned covering the syringe during the injection to conceal the allocation of intervention. For blinding of participants and personnel, three (43%) studies were of low risk of bias, three (43%) studies were of unclear risk and one (14%) study was of high risk. For blinding of outcome assessment, three (43%) studies were of low risk of bias, while four (57%) studies were of unclear risk. None of the studies had incomplete outcome data or selective reporting biases. All the studies had a low risk of other bias except for Guo 2018 [17], which mentioned that there could be a certain bias in recommending the treatment to patients due to the difference in knowledge of PRP and HA between doctors in the study.

For the cohort studies, Abate et al. [1] and Palco et al. [28] had an overall low risk of bias while Guo et al. had a moderate risk of bias (Fig. 3). For risk of confounding, Guo 2016 [18] had a serious risk of bias in the study as some bias may have existed in the selection of the technique due to the therapist’s preference and the amount of activity post-intervention was not controlled for. For bias due to deviation from intended interventions, Abate et al. [1] had a moderate risk of bias as arthrocentesis was performed when articular effusion was present, which could potentially be a form of symptomatic treatment. Bias in measurement of outcomes was unable to be assessed in the studies done by Abate et al. [1] and Palco et al. [28] as it was not mentioned if allocation was concealed to patients. All three studies had a low risk of bias in terms of selection of participants into the study, classification of interventions, missing data and selection of the reported result.

Only one study (Jacob 2017 [21]) reported on its source of funding while four studies (Abate 2015 [1], Guo 2016 [18], Jacob 2017 [21], Yu 2018 [42]) reported on any potential sources of conflict of interest.

### Meta-analysis

#### Visual Analogue Scale (VAS)

**Fig. 4:**
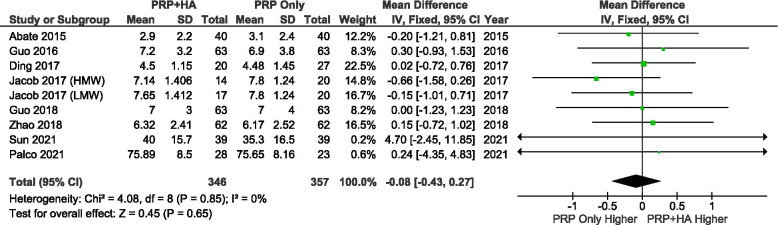
Showing the Forest plot comparing the VAS scores between PRP + HA and PRP at baseline

A total of 7 studies reported VAS scores at baseline. A random-effects model was used for the meta-analysis. The results showed that there were no significant differences in the baseline VAS scores between the groups (SMD: -0.08, 95% CI: − 0.43 to 0.27, *P* = 0.65 = n.s.) (Fig. 4).

**Fig. 5:**
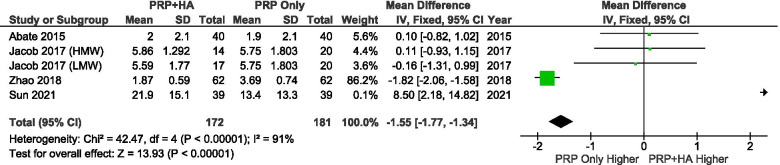
Showing the Forest plot comparing the VAS scores between PRP + HA and PRP at 4–6 weeks after treatment

A total of 3 studies reported VAS scores at 4–6 weeks after treatment. The results showed that patients who received PRP combined with HA had significantly better VAS scores than patients who received PRP alone at 4–6 weeks (SMD: -1.55, 95% CI: − 1.77 to − 1.34, *P*  < 0.00001) (Fig. 5).

**Fig. 6:**
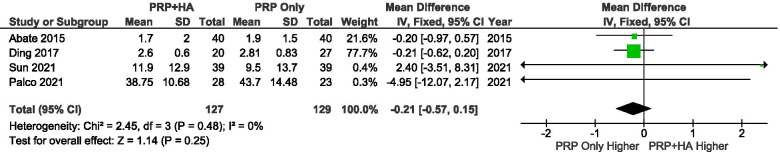
Showing the Forest plot comparing the VAS scores between PRP + HA and PRP at 3 months after treatment

A total of 2 studies reported VAS scores at 3 months after treatment. The results showed that there were no significant differences in the 3-month VAS scores between the groups (SMD: -0.21, 95% CI: − 0.57 to 0.15, *P* = 0.25 = n.s.) (Fig. 6).

**Fig. 7:**
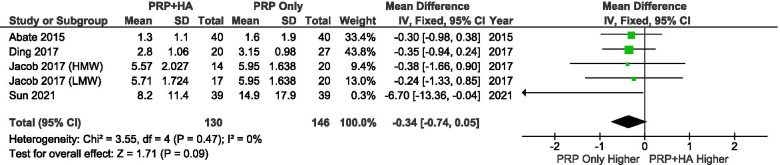
Showing the Forest plot comparing the VAS scores between PRP + HA and PRP at 6 months after treatment

A total of 4 studies reported VAS scores at 6 months after treatment. The results showed that there were no significant differences in the 6-month VAS scores between the groups (SMD: -0.34, 95% CI: − 0.74 to 0.05, *P* = 0091 = n.s.) (Fig. 7).

**Fig. 8:**
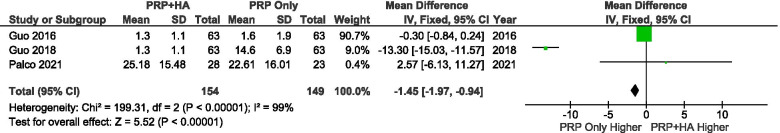
Showing the Forest plot comparing the VAS scores between PRP + HA and PRP at 12 months after treatment

A total of 2 studies reported VAS scores at 12 months after treatment. The results showed that patients who received PRP combined with HA had significantly better VAS scores than patients who received PRP alone at 12 months (SMD: -1.45, 95% CI: − 1.97 to − 0.94, *P* < 0.00001) (Fig. 8).

**Fig. 9:**
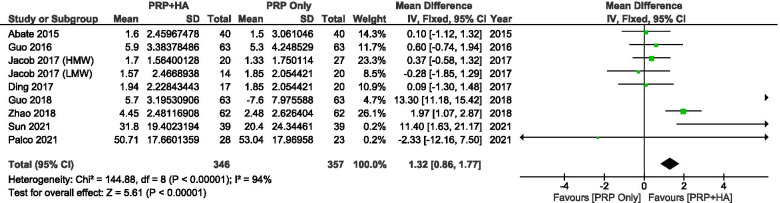
Showing the Forest plot comparing the change in VAS scores between PRP + HA and PRP post-treatment

A comparison of VAS scores between PRP combined with HA and PRP pre-treatment and post-treatment showed a significant mean improvement of − 1.32 points (95% CI: − 1.77 to − 0.86, *P* < 0.00001) (Fig. 9).

### Western Ontario and McMaster Universities Arthritis Index (WOMAC) Function score

**Fig. 10:**
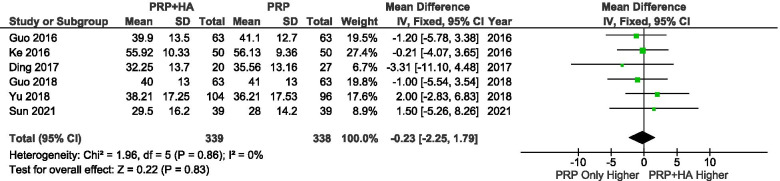
Showing the Forest plot comparing the WOMAC function scores between PRP + HA and PRP at baseline

A total of 5 studies reported the WOMAC Function Score at baseline. The results showed that there were no significant differences in the baseline WOMAC scores between the groups (SMD: -0.23, 95% CI: − 2.25 to 1.79, *P* = 0.83 = n.s.) (Fig. 10).

**Fig. 11:**
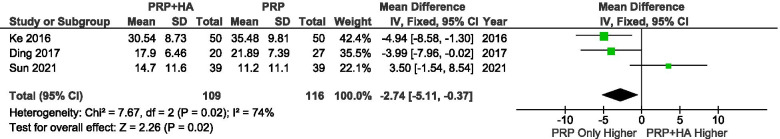
Showing the Forest plot comparing the WOMAC function scores between PRP + HA and PRP at 3 months after treatment

A total of 3 studies reported the WOMAC Function Score at 3 months after treatment. The heterogeneity in the studies is high and significant (I2 = 74%, *P* = 0.02 = n.s.), and a fixed-effects model was used for the meta-analysis. The results showed that at 3 months after treatment, the WOMAC Function Score of the group receiving PRP combined with HA was 2.74 points lower (better) than that of the group receiving PRP alone (SMD: -2.74, 95% CI: − 5.11 to − 0.37, *P* = 0.02 < 0.05) (Fig. 11).

**Fig. 12:**
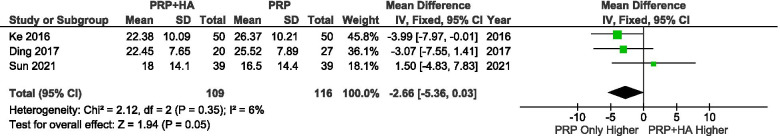
Showing the Forest plot comparing the WOMAC function scores between PRP + HA and PRP at 6 months after treatment

A total of 3 studies reported the WOMAC Function Score at 6 months after treatment. The results showed that at 6 months after treatment, the WOMAC Function Score of the group receiving PRP combined with HA was 2.66 points lower (better) than that of the group receiving PRP alone (SMD: -2.66, 95% CI: − 5.36 to 0.03, *P* = 0.05) (Fig. 12).

**Fig. 13:**
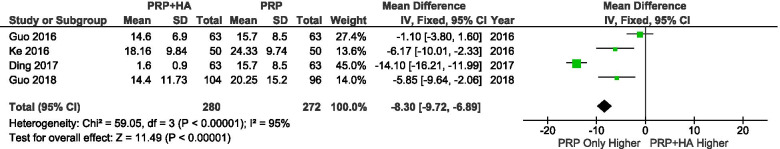
Showing the Forest plot comparing the WOMAC function scores between PRP + HA and PRP at 12 months after treatment

A total of 4 studies reported the WOMAC Function Score at 12 months after treatment. The results showed that at 12 months after treatment, the WOMAC Function Score of the group receiving PRP combined with HA was 8.30 points lower (better) than that of the group receiving PRP alone (SMD: -8.30, 95% CI: − 9.72 to − 6.89, *P* < 0.0001) (Fig. 13).

**Fig. 14:**
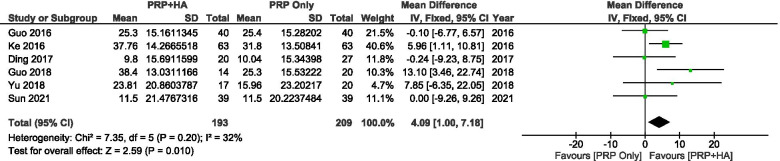
Showing the Forest plot comparing the change in WOMAC function scores between PRP + HA and PRP post treatment

A comparison of WOMAC function scores between PRP combined with HA and PRP pre-treatment and post-treatment showed a significant mean improvement of − 4.07 points (95% CI: − 7.18 to − 1.00, *P* = 0.010 < 0.05) (Fig. 14).

### International Knee Documentation Committee (IKDC) score

**Fig. 15:**
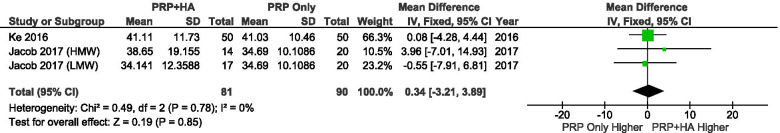
Showing the Forest plot comparing the IKDC scores between PRP + HA and PRP at baseline

A total of 3 studies reported IKDC scores at baseline. There were no significant differences in the baseline IKDC scores between the groups (SMD: 0.34, 95% CI: − 3.21 to 3.89, *P* = 0.85 = n.s.) (Fig. 15).

**Fig. 16:**
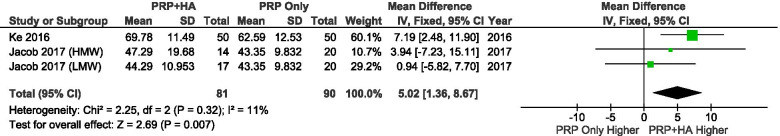
Showing the Forest plot comparing the IKDC scores between PRP + HA and PRP at 6 months after treatment

A total of 3 studies reported IKDC scores at 6 months after treatment. The results showed patients who received PRP combined with HA had significantly better IKDC scores than those who received PRP only at 6 months (SMD: 5.02, 95% CI: 1.36 to 8.67, *P* = 0.007 < 0.05) (Fig. 16).

**Fig. 17:**
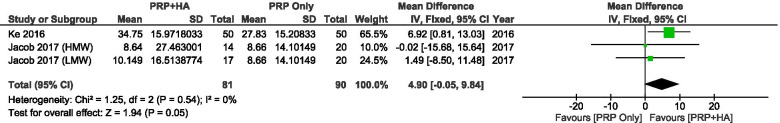
Showing the Forest plot comparing the change in IKDC scores between PRP + HA and PRP post-treatment

A comparison of IKDC scores between PRP combined with HA and PRP pre-treatment and post-treatment showed a mean improvement of − 4.90 points, which was not significant (95% CI: − 9.84 to 0.05, *P* = n.s.) (Fig. 17).

### Adverse Events (AEs)

Eight studies reported that no major adverse events such as infection occurred while 2 studies did not report the occurrence of adverse events. A total of 8 studies reported the comparison of mild AEs of PRP combined with HA and PRP alone on KOA. The main types of adverse reactions were erythema, pain, swelling, hypertension and proteinuria.

**Fig. 18:**
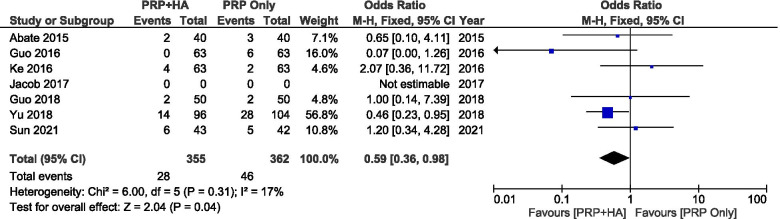
Showing the Forest plot comparing the incidence of adverse events between PRP+ HA and PRP

The results showed that PRP combined with HA was generally safer than PRP alone in terms of adverse events (SMD: 0,59, 95% CI: 0.36 to 0.98, *P* = 0.02 < 0.05) (Fig. 18).

## Discussion

The main study finding is that PRP and HA dual therapy for KOA is effective at providing pain relief and improvement in function based on improvement in VAS, IKDC and WOMAC scores up to 1 year following administration and resulted in fewer adverse events, despite a lack of large high-quality Level I studies available to be analyzed in this study.

KOA places an enormous socioeconomic burden on global healthcare systems due to its prevalence and impact. Innovative methods of treatment are constantly being sought after, with HA and PRP being two of the most notable methods in recent years. HA is a non-sulfated glycosaminoglycan and can be found naturally in articular cartilage and synovial fluid. It is responsible for lubrication and shock absorption within the joint, encourages growth and development of cartilage and bone, and it also plays an important part in regulating joint inflammation and pain caused by tissue injury [23]. In OA, intra-articular injection of HA serves to improve the viscoelasticity of the synovial fluid, reducing pain and improving joint function.

PRP, on the other hand, is made up of centrifuged whole blood, which contains higher than baseline levels of platelets. It comprises growth factors and proteins that enhance cartilage cells regeneration, promoting recovery of the joint [19]. To impede OA, PRP therapy utilizes its anti-inflammatory properties for stimulation of growth factors that encourage cartilage matrix synthesis and inhibition of innate immune response cytokines linked to cartilage erosion [10, 36]. In addition, PRP reverses the senescence process of chondrocytes, restoring the self-renewing capacity of cartilages [37].

PRP and HA combined, despite being more expensive and complicated, has been proven in some studies to have synergistic effects in promoting cartilage regeneration and reducing inflammation in OA [8]. However, this has not been extensively studied and some argue that the effects of dual therapy, considering the extra costs required, are not significantly different from that of HA or PRP alone.

Our study found better IKDC, WOMAC and VAS scores in patients who received PRP and HA dual-therapy compared to patients who received PRP-alone therapy. WOMAC function score is significantly better for the dual therapy group at 3, 6 and 12 months compared to the PRP-alone group. Additionally, there is a significant post-treatment improvement in WOMAC function scores for PRP and HA combination therapy compared to PRP monotherapy. This finding is corroborated by Lana et al. who showed that PRP and HA dual therapy resulted in an improvement in median WOMAC Pain values at 30 and 90 days compared to PRP-alone therapy [25]. In their systematic review, Zhao et al. noted that PRP and HA dual therapy achieved better WOMAC scores at 12 months compared to PRP-alone therapy [44]. However, it should be noted that there were only 3 studies available to compare WOMAC function scores at 3 and 6 months, thus limiting our ability to draw a significant conclusion from it.

Similarly, the IKDC score is significantly better for the dual therapy group at 6 months compared to the PRP-alone group. Rahman et al. showed that the mean IKDC score at 1 week, 1 month and 3 months were significantly higher for PRP and HA dual therapy compared to PRP-alone therapy [31]. However, a significant post-treatment improvement in IKDC scores was not found for PRP and HA combination therapy compared to PRP monotherapy.

Significant improvement in VAS score was also seen after 4–6 weeks and 12 months, along with a significant post-treatment improvement in WOMAC function scores for the dual therapy group compared to the PRP-alone group. A study by Rabi’u and Aliyu noted that PRP and HA dual therapy provided significant pain relief, improvement in psychological and functional capacity compared with PRP-alone therapy at 6 months [30]. The improvement in IKDC, WOMAC and VAS scores are due to the synergistic effects of PRP and HA in altering the inflammatory cytokines in chondrocyte degeneration through specific mediators such as CD44 and TGF-βRII. These effects may promote cartilage regeneration and inhibit inflammation in KOA. These processes occur from 30 to 90 days after application, which explains the 3-month improvement in WOMAC in the dual therapy group [3, 6]. PRP and HA also aid in reducing inflammation through decreased IL-6 and synoviocyte matrix metalloproteinase-13 expression, which plays a key role in cartilage matrix degradation during the development and progression of OA [38]. The lubrication and support to the extracellular matrix that HA provides appears to enable earlier benefit to the PRP injection in terms of function and pain relief, hence resulting in better rehabilitation and earlier return to daily activities [26].

VAS scores were generally higher in the PRP group, especially in the early post-injection phase. While there is no evidence in the literature to explain this phenomenon, patients often experience post-injection flare following PRP administration [15]. This is likely due to the pro-inflammatory factors in PRP. Perhaps, co-administration with HA might have a dilutional effect on some of these factors giving rise to reduced flare pain. Further basic science research is needed to prove this postulated theory.

Our study also found that the PRP-alone group had a higher incidence of AEs compared to the PRP and HA group. This could be explained by the study by Yu et al. [42] having a larger effect on the overall outcome due to the large number of reported AEs. However, most of the AEs reported by Yu are systemic complications such as proteinuria and hypertension, and are not reflective of the milder side effects such as pain and swelling reported by the other studies. While the reasons for the reduced number of local AEs with PRP and HA dual therapy have yet to be elucidated, Xu et al. [41] postulated that HA in PRP and HA dual therapy potentially attenuates leukocyte-induced oxidative stress and proteolytic enzymes and hence improving the microenvironment of the knee joint.

A barrier to the clinical applicability of injectables research is the heterogeneity in composition, preparation and administration of PRP. Our study reflected the great inter-product variation in terms of volume of blood harvested, centrifugation settings, platelet/ HA concentration, activation process, devices used and the timing of injections, which could lead to differing outcomes. Additionally, the paucity of and inconsistency in details reported regarding this makes it difficult to aggregate the data and perform an objective comparison between different studies [32, 14, 27]. As such, numerous classification systems have been developed to make the reporting process more amenable to objective analysis [24, 33, 13]. However, none has gained widespread acceptance and as such, significant heterogeneity remains. Considering the large number of studies done about injectables in KOA, standardisation of PRP and HA preparation is important in attempting to draw a meaningful conclusion about whether to incorporate PRP and HA combination therapy into the treatment plan for patients.

The findings of this study are corroborated by a systematic review by Zhao et al. [44] that favoured PRP and HA combination over PRP-alone therapy. However, a systematic review by Baria et al. [6] did not find a compelling reason for combination therapy over PRP-alone therapy. The difference in conclusions drawn is due to the fact that both studies only had 2 studies in common (Guo 2016 and Abate 2015). The study by Baria et al. [6] included studies by Lana or Jacob but did not include the studies by Yu et al. [42], Zhao et al. [45], Ding et al. [12], and Ke et al. [22] because they were not available in English. Meanwhile, our study included all the studies comparing PRP and HA combination therapy with PRP monotherapy from both studies and more recent studies by Palco et al. [28], Xu et al. [41] and Sun et al. [37] to allow for a better-informed comparison.

This study is one of the first few studies to perform a systematic review and meta-analysis comparing PRP and HA dual therapy and PRP-alone therapy as most studies compared the efficacy of PRP versus placebo [29, 35] or PRP versus HA [34, 40, 16]. In addition, the quality of the evidence in this study is good as all the studies included were of low to moderate risk of bias. Nonetheless, some limitations are present. Firstly, the small number of studies (*n* = 9) limited the power of conclusions drawn from the study. Secondly, a funnel plot could not be done to assess publication bias due to a small number of studies. Thirdly, the PRP concentrations used in the literature included vary from study to study, which may have had an impact on the efficacy of treating KOA. Fourthly, the included studies examined different outcomes at different time points, resulting in a small number of studies examining a certain outcome at a certain time point. Hence, this restricts our ability to draw significant conclusions at specific times. Although it could be seen as a strength of this systematic review, another possible limitation of this paper could arise in the form of the inclusion of several Chinese papers as these cannot be used for confirmation to the non-Chinese speaking researchers. We also acknowledge that the total number of studies for meta-analysis may be artificially increased as we have utilised Jacob [21] as two separate entries (LMW HA and HMW HA). Lastly, the effects of HA alone were not explored, which could provide a more holistic perspective on KOA treatment.

More high-quality RCTs are needed to avoid potential bias in selection, performance and attrition, hence increasing statistical reliability in future systematic reviews and meta-analyses. Additionally, as most of the studies in this study reported outcomes up to 12 months, a systematic review and meta-analysis can be done with longer-term studies to elucidate the efficacy of PRP and HA dual-therapy compared to PRP-alone beyond 12 months. Further studies can also be conducted on whether the ratio of PRP: HA affects the rheological properties and hence improvement in function and reduction in pain.

## Conclusion

While there is a paucity of large high-quality Level I studies, current best evidence suggests that dual therapy with PRP and HA for KOA may be effective at providing pain relief and improvement in function up to 1 year following administration.

## Abbreviations

KOA: Knee Osteoarthritis
HA: Hyaluronic Acid
PRP: Platelet-Rich Plasma
RCT: Randomised Controlled Trial
WOMAC: Western Ontario and McMaster Universities Arthritis Index
IKDC: International Knee Documentation Committee
VAS: Visual Analogue Scale
MD: Mean Difference
CI: Confidence Interval
SMD: Standard Mean Difference

## References

[1] Abate M, Verna S, Schiavone C, Di Gregorio P, Salini V (2015). Efficacy and safety profile of a compound composed of platelet-rich plasma and hyaluronic acid in the treatment for knee osteoarthritis (preliminary results). Eur J Orthop Surg Traumatol.

[2] Anandacoomarasamy A, March L (2010). Current evidence for osteoarthritis treatments. Ther Adv Musculoskelet Dis.

[3] Andia I, Abate M (2014). Knee osteoarthritis: hyaluronic acid, platelet-rich plasma or both in association?. Expert Opin Biol Ther.

[4] Altman R, Bedi A, Manjoo A, Niazi F, Shaw P, Mease P (2018). Anti-inflammatory effects of intra-articular hyaluronic acid: a systematic review. Cartilage.

[5] Alves R, Grimalt R (2017). A review of platelet-rich plasma: history, biology, mechanism of action, and classification. Skin Appendage Disord.

[6] Baria M, Vasileff W, Borchers J, DiBartola A, Flanigan D, Plunkett E, Magnussen R (2021) Treating knee osteoarthritis with platelet-rich plasma and hyaluronic acid combination therapy: a systematic review. Am J Sports Med 363546521998010 10.1177/0363546521998010 33831332

[7] Campbell K, Saltzman B, Mascarenhas R, Khair M, Verma N, Bach B, Cole B (2015). Does intra-articular platelet-rich plasma injection provide clinically superior outcomes compared with other therapies in the treatment of knee osteoarthritis? A systematic review of overlapping Meta-analyses. Arthroscopy.

[8] Chen W, Lo W, Hsu W, Wei H, Liu H, Lee C, Tina Chen S, Shieh Y, Williams D, Deng W (2014). Synergistic anabolic actions of hyaluronic acid and platelet-rich plasma on cartilage regeneration in osteoarthritis therapy. Biomaterials.

[9] Cui A, Li H, Wang D, Zhong J, Chen Y, Lu H (2020). Global, regional prevalence, incidence and risk factors of knee osteoarthritis in population-based studies. EClinicalMedicine.

[10] Daheshia M, Yao J (2008). The interleukin 1β pathway in the pathogenesis of osteoarthritis. J Rheumatol.

[11] Dhurat R, Sukesh M (2014). Principles and methods of preparation of platelet-rich plasma: a review and author′s perspective. J Cutan Aesthet Surg.

[12] Ding Q, Lv S, Shen X, Tong P (2017). A prospective randomized controlled study on platelet-rich plasma (PRP) combined with sodium hyaluronate (HA) intra-articular injection in the treatment of knee osteoarthritis. Shanghai Med Pharmaceut J.

[13] Dohan Ehrenfest David M (2014). Classification of platelet concentrates (platelet-rich plasma-PRP, platelet-rich fibrin-PRF) for topical and infiltrative use in orthopedic and sports medicine: current consensus, clinical implications and perspectives. Muscles Ligaments Tendons J.

[14] Fadadu P, Mazzola A, Hunter C, Davis T (2019). Review of concentration yields in commercially available platelet-rich plasma (PRP) systems: a call for PRP standardization. Reg Anesthesia Pain Med.

[15] Filardo G, Di Matteo B, Di Martino A, Merli M, Cenacchi A, Fornasari P, Marcacci M, Kon E (2015). Platelet-rich plasma intra-articular knee injections show no superiority versus viscosupplementation. Am J Sports Med.

[16] Filard G, Kon E, Di Martino A, Di Matteo B, Merli M, Cenacchi A, Fornasari P, Marcacci (2012). Platelet-rich plasma vs hyaluronic acid to treat knee degenerative pathology: study design and preliminary results of a randomized controlled trial. BMC Musculoskelet Disord.

[17] Guo Y, Yu H, Yong M (2018). Treatment of knee osteoarthritis with mixture of platelet-rich-plasma plus hyaluronic acid. Chin J Joint Surg (Electronic Edition).

[18] Guo Y, Yu H, Yuan L (2016). Treatment of knee osteoarthritis with platelet-rich plasma plus hyaluronic acid in comparison with platelet-rich plasma only. Int J Clin Exp Med.

[19] Hermans J, Bierma-Zeinstra S, Bos P, Niesten D, Verhaar J, Reijman M (2019). The effectiveness of high molecular weight hyaluronic acid for knee osteoarthritis in patients in the working age: a randomised controlled trial. BMC Musculoskelet Disord.

[20] Hsu H. Knee Osteoarthritis. StatPearls. https://www.ncbi.nlm.nih.gov/books/NBK507884/. Published June 29, 2020. Accessed 15 Jan 2021

[21] Jacob G, Shetty V, Shetty S (2017). A study assessing intra-articular PRP vs PRP with HMW HA vs PRP with LMW HA in early knee osteoarthritis. J Arthrosc Jt Surg.

[22] Ke C, Zhang R, Xue J (2016). Clinical efficacy of autologous platelet-rich plasma combined with intra-articular hyaluronic acid injection for knee osteoarthritis. Chin Gen Pract.

[23] Kennedy M, Whitney K, Evans T, LaPrade R (2018). Platelet-rich plasma and cartilage repair. Curr Rev Musculoskelet Med.

[24] Kon E, Di Matteo B, Delgado D, Cole B, Dorotei A, Dragoo J, Filardo G, Fortier L, Giuffrida A, Jo C, Magalon J, Malanga G, Mishra A, Nakamura N, Rodeo S, Sampson S, Sánchez M (2020). Platelet-rich plasma for the treatment of knee osteoarthritis: an expert opinion and proposal for a novel classification and coding system. Expert Opin Biol Ther.

[25] Lana H, Weglein A, Sampson S, Vicente E, Huber S, Souza C, Ambach M, Vincent H, Urban-paffaro A, Onodera C, Annichino-bizzacchi J, Santana M, Belangero W (2016). Randomized controlled trial comparing hyaluronic acid, platelet-rich plasma and the combination of both in the treatment of mild and moderate osteoarthritis of the knee. J Stem Cells Regen Med.

[26] Mautner K, Malanga G, Smith J, Shiple B, Ibrahim V, Sampson S, Bowen J (2015). A call for a standard classification system for future biologic research: the rationale for new PRP nomenclature. PM&R.

[27] Moussa M, Lajeunesse D, Hilal G, El Atat O, Haykal G, Serhal R, Chalhoub A, Khalil C, Alaaeddine N (2017). Platelet rich plasma (PRP) induces chondroprotection via increasing autophagy, anti-inflammatory markers, and decreasing apoptosis in human osteoarthritic cartilage. Exp Cell Res.

[28] Palco M, Fenga D, Basile GC, Rizzo P, Cavalieri B, Leonetti D (2021). Platelet-rich plasma combined WITH hyaluronic ACID versus leucocyte and platelet-rich plasma in the conservative treatment of knee osteoarthritis. A retrospective study. Medicina.

[29] Patel S, Dhillon M, Aggarwal S, Marwaha N, Jain A (2013). Treatment with platelet-rich plasma is more effective than placebo for knee osteoarthritis. Am J Sports Med.

[30] Rabi’u M, Aliyu D (2019). Effect of addition of hyaluronic acid on platelet rich plasma in treatment of chronic osteoarthritis. EAS J Orthop Physiother.

[31] Rahman M, Islam S, Hossain M, Arefin M, Islam M, Begum S, Shompa S, Akhtaruzzaman A (2019). Effects of platelet rich plasma in combination with hyaluronic acid in the treatment of primary knee osteoarthritis. J Natl Inst Neurosci Bangladesh.

[32] Rodeo S (2019). The need for minimum reporting standards for studies of “biologics” in sports medicine. Am J Sports Med.

[33] Rossi L, Murray I, Chu C, Muschler G, Rodeo S, Piuzzi N (2019). Classification systems for platelet-rich plasma. Bone Joint J.

[34] Sánchez M, Fiz N, Azofra J, Usabiaga J, Aduriz Recalde E, Garcia Gutierrez A, Albillos J, Gárate R, Aguirre J, Padilla S, Orive G, Anitua E (2012). A randomized clinical trial evaluating plasma rich in growth factors (PRGF-Endoret) versus hyaluronic acid in the short-term treatment of symptomatic knee osteoarthritis. Arthroscopy.

[35] Smith P (2016). Intra-articular autologous conditioned plasma injections provide safe and efficacious treatment for knee osteoarthritis. Am J Sports Med.

[36] Sowers M, Karvonen-Gutierrez C (2010). The evolving role of obesity in knee osteoarthritis. Curr Opin Rheumatol.

[37] Sun SF, Lin GC, Hsu C (2021). Comparing efficacy of intraarticular single crosslinked Hyaluronan (HYAJOINT Plus) and platelet-rich plasma (PRP) versus PRP alone for treating knee osteoarthritis. Sci Rep.

[38] Sundman E, Cole B, Karas V, Della Valle C, Tetreault M, Mohammed H, Fortier L (2013). The anti-inflammatory and matrix restorative mechanisms of platelet-rich plasma in osteoarthritis. Am J Sports Med.

[39] Tamer T (2013). Hyaluronan and synovial joint: function, distribution and healing. Interdiscip Toxicol.

[40] Vaquerizo V, Plasencia M, Arribas I, Seijas R, Padoilla S, Orive G, Anitua E (2013). Comparison of intra-articular injections of plasma rich in growth factors (PRGF-Endoret) versus durolane hyaluronic acid in the treatment of patients with symptomatic osteoarthritis: a randomized controlled trial. Arthroscopy.

[41] Xu Z, He Z, Shu L, Li X, Ma M, Ye C (2021). Intra-articular platelet-rich plasma combined with hyaluronic acid injection for knee osteoarthritis is superior to platelet-rich plasma or hyaluronic acid alone in inhibiting inflammation and improving pain and function. Arthroscopy: J Arthrosc Related Surg.

[42] Yu W, Xu P, Huang G, Liu L (2018). Clinical therapy of hyaluronic acid combined with platelet-rich plasma for the treatment of knee osteoarthritis. Exp Ther Med.

[43] Zhang Y, Jordan J (2010). Epidemiology of osteoarthritis. Clin Geriatr Med.

[44] Zhao J, Huang H, Liang G, Zeng L, Yang W, Liu J (2020). Effects and safety of the combination of platelet-rich plasma (PRP) and hyaluronic acid (HA) in the treatment of knee osteoarthritis: a systematic review and meta-analysis. BMC Musculoskelet Disord.

[45] Zhao X (2018). Clinical effect of sodium hyaluronate injection combined with autologous platelet rich plasma injection for knee osteoarthritis. Clin Res Pract.

